# The Trends of Medical Care Expenditure with Adjustment of Lifestyle Habits and Medication; 10-Year Retrospective Follow-Up Study

**DOI:** 10.3390/ijerph17249546

**Published:** 2020-12-20

**Authors:** Haruko Ono, Kotomi Akahoshi, Michiaki Kai

**Affiliations:** 1 Department of Community Health Nursing, Oita University of Nursing and Health Science, Oita 870-1201, Japan; akahoshi@oita-nhs.ac.jp; 2 Department of Environmental Health Science, Oita University of Nursing and Health Science, Oita 870-1201, Japan; kai@oita-nhs.ac.jp

**Keywords:** medical care expenditure, lifestyle habits, specific health checkups

## Abstract

In Japan, the prevention of lifestyle-related diseases is the most important issue for the optimization of medical expenditure. This study aimed to analyze the impact of lifestyle and medication status on medical expenditure. Health checkup data and medical expenditure records of a retrospective cohort of 1463 people aged between 40 and 65 years old who underwent specific health checks at least three times between 2008 and 2017 were analyzed. Regression analysis was performed with medical expenditure as the dependent variable and age, gender, waist ratio, medication status, and lifestyle habits as independent variables using a Tobit model. Focusing on the factors that increase medical expenditure, the regression coefficients of age, medication status, weight gain of 10 kg or more since the age of 20, and walking more than 1 h per day were 0.048 (95% CI 0.04 to 0.06), 1.020 (95% CI 0.88 to 1.16), 0.210 (95% CI 0.06 to 0.36), and −0.208 (95% CI −0.35 to −0.07), respectively. The estimate of 5-year cumulative medical expenditure showed that those with walking habits without medication had the lowest medical expenditure. The result of this study suggests that walking more than 1 h a day may lower health expenditure in the general population.

## 1. Introduction

Obesity, excessive alcohol consumption, and smoking have been reported to increase the risk of lifestyle-related diseases such as heart disease, diabetes, cerebrovascular disease, and malignancies, which can lead to high medical care expenditure [1,2,3,4]. It is estimated that the global health care expenditure for noncommunicable diseases, including lifestyle-related diseases as well as psychiatric disorders, will be $47 trillion in the next 2 decades, which represents a significant financial burden worldwide [5].

In Japan, it is reported that the national health care expenditure for the 2017 fiscal year was USD 382 billion (JPY 40 trillion), of which more than 35% was used for lifestyle-related diseases [6]. Prevention of lifestyle-related diseases is the most important issue for the optimization of medical expenditure. The Japanese government introduced specific health checkups and health guidance in 2008 that aimed for early detection of metabolic syndrome and a reduction in medical expenditure [7].

Previous studies have reported that intervention to improve lifestyle habits by reducing weight and waist circumference (WC) can decrease the risk and the prevalence of diabetes [8,9,10,11,12,13,14]. These interventions were conducted for less than 1 year and indicated effectiveness in the short term.

This study reported the relationship between 10-year changes in waist circumference and lifestyle habits. The long-term follow-up study suggested that the average body weights for both men and women were significantly reduced over the 10-year period while WC increased regardless of lifestyle habits. Moreover, an increase in waist circumference was associated with worse estimated levels of triglyceride, low-density lipoprotein cholesterol, and high-density lipoprotein cholesterol after 10 years [15].

In promoting the optimization of medical-care cost, it is necessary to consider not only individuals with metabolic syndrome, but also healthy people.

This study analyzed the medical expenditure for people who were healthy and those with metabolic syndrome, using checkups and health insurance claims data from residents who regularly attended specific health checkups between 2008 and 2017. Based on the results, we quantitatively assessed the impact of changes over the 10-year period on medical expenditure.

## 2. Japanese Insurance System

The Japanese government has organized several health insurance systems, and all citizens are obliged to join one. One of them, the National Health Insurance, covers 35% of the Japanese population, mainly farmers, self-employed or retired people, and nonworking dependents under the age of 75. Access to medical care is unlimited for all persons. The unit price of medical care is determined by the government. Individuals have to pay 10 to 30% of their medical expense, with the rest covered by insurance. In the present study, medical expenditure refers to the medical expense paid individually.

## 3. Methods

### 3.1. Participants

A population-based longitudinal and individualized observational study was conducted using the specific health checkup data of the residents registered in the National Health Insurance of a city in 2008. The cohort consisted of 6621 residents. The data were annually collected and collated from 2008 to 2017. We focused on 2984 residents who were between 40 and 65 years old, and we excluded residents over 65 years old in 2008 and residents who could not follow up until 2017. Of these, 1474 residents who had received specific health checkups at least three times (in 2008, 2012–2013, and 2017) were included in the analysis.

In Japan, the average annual medical expenditure for outpatients is USD 3273 (JPY 342,000) per person, which corresponds to USD 16,365 (JPY 1.71 million) in 5 years [6]. In this survey, in order to investigate the relationship between lifestyle habits and medical expenditure, we excluded 11 subjects whose cumulative medical expenditure for 5 years exceeded USD 57,422 (JPY 6 million). Their medical expenditure was due to long-term treatment for schizophrenia and medication for hepatitis as well as rheumatism. We included 1463 subjects in the final analysis.

### 3.2. Collection of Clinical, Anthropometric, and Lifestyle Information

Anthropometric measurements of individuals wearing light clothing and no shoes were conducted by well-trained examiners. Body weight (BW) was measured in an upright position to the nearest 0.1 kg. Body mass index (BMI) was calculated by dividing BW (kg) by height squared (m^2^). Waist circumference (WC) measurements were obtained after normal respiration and were measured to the nearest 0.1 cm at the umbilical level (the standard Japanese method) using a flexible anthropometric tape. Self-reported questionnaires regarding patients’ lifestyle habits based on a “specific health examination” were used for data collected by health authorities. The questionnaire included questions on medications (hypertension, diabetes mellitus, and hyperlipidemia), smoking history (≥100 cigarettes in the past year), weight gain of over 10 kg since the age of 20, regular exercise (at least 30 min, three times a week), walking more than 1 h every day, intake of alcohol nearly every day, eating an evening meal within 2 h of going to bed, and skipping breakfast. Respondents chose “yes” or “no” for easier understanding.

### 3.3. Medical Expenditure

The subjects were linked to monthly medical expenditure records with bill dates from April 2013 through March 2017. The records of medical expenditure included fees for outpatient services, medications, and hospitalization, excluding dental consultations and treatments. To evaluate lifestyle-related medical care costs, we calculated the cumulative medical expenditure by adding the outpatient fees and the medication cost for 5 years per person. Medical expenditure was presented in USD (JPY) (JPY 104.49 = USD 1 as of 10 December 2020).

### 3.4. Statistical Analysis

Data of all continuous variables were expressed as the mean and SD while categorical variables were expressed as numbers and percentages. When comparing the two groups, χ2 tests were used for categorical variables and Welch two-sample t-tests were used for continuous variables.

The medical costs were zero-truncated (non-negative) values, and an individual expenditure of 0 yen could not be ignored. In addition, medical costs tended towards right distorted distribution. The Tobit model was used when the dependent variable was represented by truncated data and distorted distribution, as were medical costs. We analyzed the data using the Tobit model in this study as well. The analysis was performed with medical cost as the dependent variable and age, sex, waist ratio, status of medication, and lifestyle habits as the independent variables. Only the effects of age, sex, waist ratio, and baseline waist circumference on cumulative medical expenditure were examined in Model I. In Model II, medication states and lifestyle habits were added to Model I as independent variables. The Tobit analysis used the same variables both for groups taking medication and groups not taking medication.

These analyses were conducted using R software (R Foundation for statistical Computing, Vienna, Austria) [16]. A *p*-value of <0.05 was considered to be statistically significant for all of the analyses.

### 3.5. Ethics Statement

This study was approved by the Research Ethics Safety Committee of Oita University of Nursing and Health Sciences before implementation (registration number 18–69). The study fell under the category of “ethical guidance related to epidemiological surveys” since it used health data. The health checkup data received from the city did not include any information that could identify the participating individuals. There was no negative impact on the participants whether they agreed or declined to participate in the study, and there were no issues concerning the protection of human rights.

## 4. Results

There were 1463 subjects, with an average age of 68.1 years in 2017. The average age of the 451 males was 67.2 years old, and that of the 1012 females was 68.5 years old. The mean and median cumulative total medical costs over 5 years were USD 12,161 (JPY 1,270,720) for the males and USD 8143 (JPY 850,820) for the females. Eleven people had 0 yen in expenditure.

The characteristics of the subjects in 2017 by the change in the WC ratio are shown in Table 1. There were significant differences in age, the percentage of the men, WC, BMI, smoking status, and weight gain from age 20 when looking at the entire group. There were significant differences in age, the percentage of the men, WC, and BMI among those who were taking medication, and there were significant differences in the percentage of the men, WC, BMI, and smoking status among those without medication. The cumulative medical costs per person for 5 years were USD 12,513 (JPY 1,307,470) in the WC increase group and USD 12,018 (JPY 1,255,720) in the WC decrease group. Among those who were taking medications, the cost for the WC increase group was USD 11,496 (JPY 1,201,180), and the cost for the WC decrease group was UDS 11,886 (JPY 1,242,020). For those not taking medications, the cost for the WC increase group was USD 5759 (JPY 601,740) and the cost for the WC decrease group was USD 5304 (JPY 554,190).

Table 2 reports the results of the Tobit analyses, which identified the effects of waist ratio and lifestyle habits on medical expenditure in all subjects. In Model I, age (*p* < 0.001), waist ratio (*p* = 0.009), and baseline waist circumference (*p* = 0.009) had a significant positive effect on medical expenditure.

Multicollinearity was assessed by using Variance Inflation Factor (VIF) in Model II. Each VIF value was 1.08 for age, 1.16 for sex, 1.07 for waist ratio, 1.45 for baseline waist circumference, 1.10 for taking medication, 1.26 for 10 kg of weight gain since the age of 20, and 1.02 for walking 1 h every day. These were moderately correlated. In order to eliminate multicollinearity as much as possible, the possibly correlated baseline waist circumference (1.45) was excluded in Model II.

In Model II, age (*p* < 0.001), taking medications (*p* < 0.001), and an increase of 10 kg since the age of 20 (*p* = 0.007) had a significantly positive effect on medical expenditure while walking for more than an hour daily (*p* = 0.003) had a significantly negative effect.

Table 3 reports the results of the Tobit analyses, which identified the effects of waist ratio and lifestyle habits on medical expenditure by medication status. For the medication group, age (*p* = 0.000), treatment for diabetes (*p* = 0.000), and an increase of 10 kg since the age of 20 (*p* = 0.012) had a significant positive effect on medical costs. For the nonmedication group, age (*p* = 0.000) had a significant positive effect while sex (*p* = 0.005) and walking for more than an hour daily (*p* = 0.005) had a significant negative effect on medical costs.

**Figure 1:**
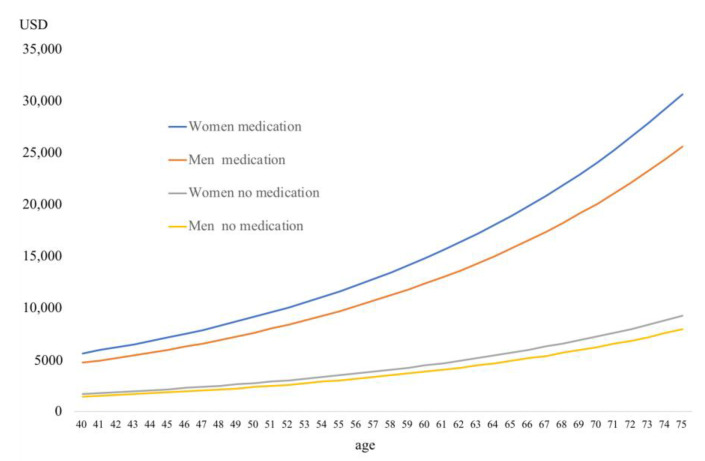
Five-year cumulative outpatient and medication expenditure by medication status.

**Figure 2:**
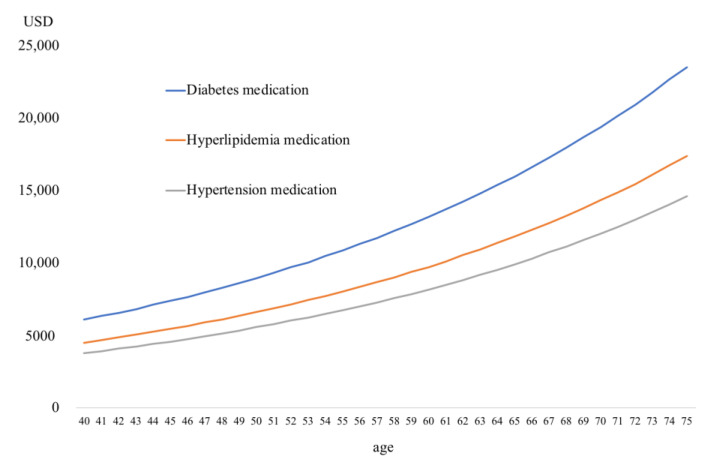
Five-year cumulative outpatient and medication expenditure for men by medication type.

**Figure 3:**
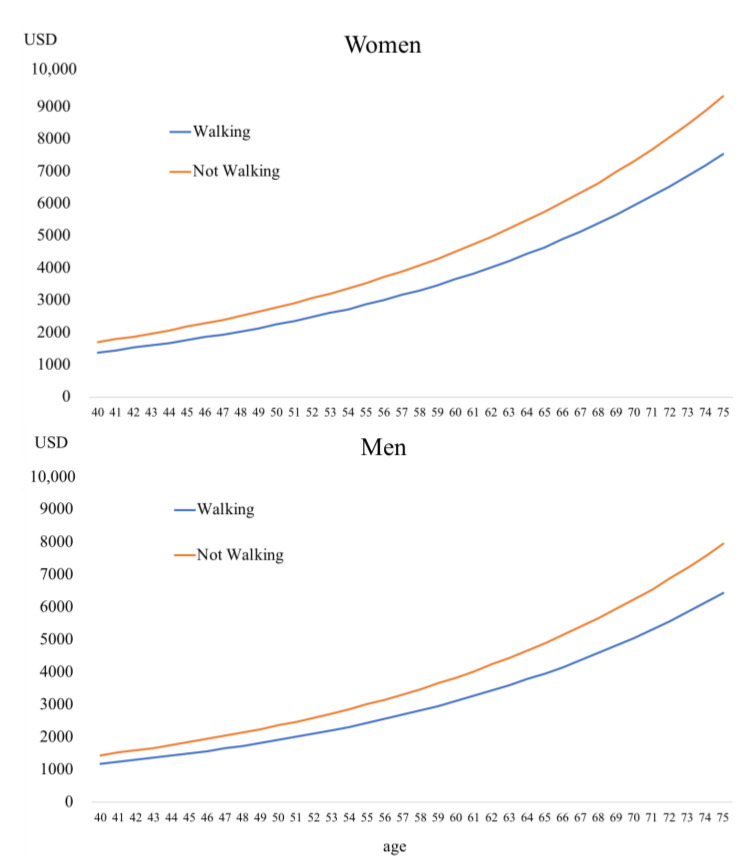
Five-year cumulative outpatient and medication expenditure for subjects without medication.

Figure 1, Figure 2 and Figure 3 show 5-year cumulative outpatient and medication expenditure based on the results of the Tobit analyses.

Figure 1 shows that 5-year cumulative medical expenditure was high for women taking medication, about USD 5642 (JPY 589,515) for women 40 years old and about USD 30,694 (JPY 3.2 million) for women 75 years old. On the other hand, for women not taking medicine, the expenditure was about USD 1698 (JPY 177,377) for women 40 years old and about USD 9235 (JPY 965,011) for women 75 years old.

Figure 2 shows 5-year cumulative medical expenditure for men by medication type. There was no significant difference in medical expenditure. Medical expenditure for diabetes medication was the highest at about USD 6093 (JPY 599,715) for men 40 years old and about USD 23,533 (JPY 2.5 million) for men 75 years old.

Figure 3 shows 5-year cumulative medication expenditure for subjects without medication by walking habits. Men with walking habits had the lowest medical expenditure, at about USD 1184 (JPY 123,764) for men 40 years old and about USD 6444 (JPY 673,335) for men 75 years old.

## 5. Discussion

This study focused on the changes in both waist circumference after 10 years and medical expenditure after 5 years by lifestyle habits in the same population-based cohort. This study indicated that the increase in medical expenditure was strongly dependent on age and medication status; inversely, the habit of walking for 1 h or more a day had a mitigating effect. In particular, those taking medication were affected by types of medication for diabetes and dyslipidemia and 10 kg of weight gain since the age of 20. Even among those not taking medication, medical expenditure increased with age. In contrast, males and those having a habit of walking for more than 1 h a day had a lowered medical expenditure.

Several studies have reported a positive association between obesity and medical expenditure. One study estimated that obesity increased individual medical care costs by USD 3429 a year in 2013 in the United States [17]. A two-part model estimated that adult obesity raised annual medical care costs by USD 3508 per obese individual, and medical care expenditure increased as BMI increased [18].

A Generalized Linear Model (GLM) analysis estimated that the medical expenditure of obese individuals with BMI >30 kg/m^2^ was 1.21–1.40 times higher than that of normal-weight individuals in Korea [19]. Compared to individuals without diabetes, individuals with diabetes had a higher medical expenditure; the bulk of the expenditure came from medication and hospitalization [20,21]. In addition, annual medical expenditure was significantly higher for diabetic patients with obesity than those without obesity [18,22,23]. In Japan, the obesity-related cardiovascular risk factor that greatly attributed to medical expenditure was hypertension, independent of abdominal obesity [24]. In this study, the increase in medical expenditure was associated with taking medication for lifestyle-related diseases, especially diabetes. Against our expectations, the changes in waist circumference did not directly affect the marked increase in medical expenditure. The weight gain of 10 kg since the age of 20 affected medical expenditure. Medical expenditure was influenced by a change in lifestyle habits over a long period of about 40 years, considering the average age of 68 among the subjects.

In Japan, since the Act on Assurance of Medical Care for Elderly People was introduced in 2008, specific health checks have been conducted to prevent and detect metabolic syndrome early in order to lower medical expenditure. In the implementation of the act, health guidance has been provided to those at risk but not to those taking medications for lifestyle-related diseases [25]. The results of this study suggest that even those taking medications need health guidance to control weight gain after the age of 20.

Many studies have investigated the effects of physical activity on medical expenditure by simulation [26,27] and direct estimation [28,29,30,31,32]. In Japan, it has been reported that a reduction in medical expenditure was associated with walking [33,34,35]. In that study, compared to individuals walking less than 0.5 h a day, the reductions in medical expenditure per month were USD 6.7–24.9 (JPY 700–2600) more for individuals walking 0.5–1 h a day and more than 1 h a day. Assuming that walking speed is 67 m/min and the length of one step is 70 cm, the reduction in medical expenditure was estimated at USD 0.000052 (JPY 0.0054) per step. In this study, the medical expenditure of individuals walking over 1 h a day at the age of 60 was USD 3118 (JPY 325,806), compared to USD 3847 (JPY 401,938) for those without the habit of walking, a reduction of about USD 12 (JPY 1300) in 1 month. It is known that physical activity decreases both mortality rate and disability [36,37] and that walking leads to a lower medical expenditure [33,34,35,38]. For example, walking has been significantly associated with reduced risk of cardiovascular disease [39], stroke [40], coronary heart disease [41], type 2 diabetes [33], and hypertension [42]. As a result, walking also reduces expenditure on medication needed for these conditions [43].

### Study Limitations

There were several limitations to this study. First, the study was limited to middle-aged and older participants in a relatively small area, so selection bias needs to be considered. In addition, per capita health care costs were higher in our study area than in Japan as a whole. Second, the participants were limited to those who had regular health checkups, which may indicate a high level of health awareness. Third, the variables of lifestyle habits that this study used were the same as those of the Japanese government’s questionnaire for specific medical examinations. Therefore, lifestyle habits were analyzed using binary variables. Statistical significance was not achieved when compared to continuous variables such as smoking, 10 kg of weight gain since the age of 20, exercise habits, and alcohol intake. In the future, it will be necessary to conduct an analysis using indicators that can evaluate lifestyle habits in more detail. Fourth, we were unable to classify medical expenditure by each disease due to the nature of the medical expenditure records. Further research is needed to determine the association between each disease and its health care expenditure. Finally, since this study was conducted in a limited region in Japan, it is necessary to consider this limitation for generalization.

## 6. Conclusions

The results of this study showed that age, medication for lifestyle-related disease, and weight gain since the age of 20 are likely to be associated with higher health care expenditure in the general population. Moreover, this study was consistent with previous studies in that walking over 1 h a day was associated with lower health care costs in the general population.

## Figures and Tables

**Table 1:** Characteristics of the subjects by medication status at the end of the follow-up period.

	All Subjects (n = 1463)			Medication (n = 744)			No Medication (n = 719)		
	Increased Waist Ratio	Decreased Waist Ratio		Increased Waist Ratio	Decreased Waist Ratio		Increased Waist Ratio	Decreased Waist Ratio	
Number (%)	424 (29.0)	1039 (71.0)		231 (31.0)	513 (69.0)		193 (26.8)	526 (73.2)	
Age	64.3 ± 6.2	63.5 ± 5.7	0.022	64.8 ± 5.3	65.6 ± 4.7	0.037	66.1 ± 6.8	67.1 ± 6.3	0.071
Men (%)	104 (24.5)	347 (33.4)	0.002	66 (28.6)	167 (32.6)	0.000	38 (19.7)	180 (34.2)	0.002
WC	87.4 ± 9.6	81.9 ± 9.2	0.000	89.7 ± 9.3	84.2 ± 9.1	0.000	84.7 ± 9.2	79.8 ± 8.8	0.000
BMI	23.6 ± 3.6	22.6 ± 3.3	0.000	24.4 ± 3.6	23.4 ± 3.4	0.001	22.7 ± 3.4	21.9 ± 3.1	0.002
SBP	128.5 ± 20.0	129.0 ± 18.0	0.630	131.4 ± 17.3	131.0 ± 16.8	0.762	125.5 ± 18.1	127.0 ± 19.0	0.189
DBP	75.3 ± 10.9	74.7 ± 10.7	0.301	76.5 ± 10.2	75.4 ± 10.8	0.171	73.9 ± 11.5	74.0 ± 11.0	0.940
TG	112.3 ± 59.8	110.1 ± 60.9	0.528	118.8 ± 61.1	117.7 ± 60.1	0.819	104.5 ± 57.3	102.7 ± 60.9	0.714
LDL	127.4 ± 30.2	125.7 ± 29.0	0.311	121.0 ± 28.0	117.9 ± 27.5	0.167	135.2 ± 31.0	133.3 ± 28.5	0.457
HDL	67.6 ± 16.6	66.9 ± 17.5	0.473	64.8 ± 15.4	64.3 ± 16.8	0.636	70.8 ± 17.4	69.4 ± 17.9	0.342
Smoking (%)	33 (7.8)	123 (11.8)	0.029	20 (8.7)	55 (10.7)	0.463	13 (6.7)	68 (12.9)	0.028
Weight gain (%)	153 (36.1)	309 (29.7)	0.022	99 (42.9)	187 (36.5)	0.130	54 (28.0)	122 (23.2)	0.213
Regular exercise (%)	180 (42.5)	466 (44.9)	0.416	97 (42.0)	240 (46.8)	0.216	83 (43.0)	226 (43.0)	1.000
Walking (%)	201 (47.4)	505 (48.6)	0.683	111 (48.1)	245 (47.8)	1.000	90 (46.6)	260 (49.4)	0.582
Late-night eating (%)	52 (12.3)	117 (11.3)	0.657	29 (12.6)	61 (11.9)	0.921	23 (11.9)	56 (10.6)	0.719
Skipping breakfast (%)	39 (9.2)	91 (8.8)	0.865	26 (11.3)	40 (7.8)	0.172	13 (6.7)	51 (9.7)	0.289
Drinking alcohol (%)	72 (17.0)	206 (19.8)	0.143	39 (16.9)	103 (20.1)	0.419	33 (17.1)	103 (19.6)	0.396
Medical expenditure	USD 12,513	USD 12,018	0.634	USD 11,496	USD 11,886	0.862	USD 5759	USD 5304	0.615

Characteristics are expressed as the mean ± SD for continuous variables and n (%) for categorical variables. *p*-values were derived from paired t-tests for continuous variables and χ2 tests for the categorical variables of waist circumference (WC), diastolic blood pressure (DBP), systolic blood pressure, (SBP), triglycerides (TG), low-density lipoprotein cholesterol (LDL-C), high-density lipoprotein cholesterol (HDL), smoking (smoking status), weight gain (10 kg of weight gain since the age of 20), walking (walking 1 h every day), late-night eating (eating an evening meal late at night), and medical expenditure (cumulative medical expenditure).

**Table 2:** Results of the Tobit regression analyses with individual 5-year cumulative outpatient and medication expenditure as the dependent variable.

Dependent Variable	Independent Variables	Model I				Model II			
		B	SE	95%CI	*p*-Values	B	SE	95%CI	*p*-Values
Cumulative medical expenditure	Intercept	6.8722	0.3906	6.11–7.64	<0.001	7.5576	0.372	6.83–8.29	<0.001
	Age	0.0664	0.0065	0.05–0.08	<0.001	0.0484	0.006	0.04–0.06	<0.001
	Sex	−0.1635	0.0868	−0.33–0.01	0.060	−0.1814	0.079	−0.34–−0.03	0.054
	Waist ratio	0.2188	0.0839	0.05–0.38	0.009	0.0932	0.079	−0.06–0.25	0.340
	Baseline waist circumference	0.2348	0.0896	0.06–0.41	0.009				
	Taking medication					1.0200	0.073	0.88–1.16	<0.001
	10 kg of weight gain since the age of 20					0.2102	0.078	0.06–0.36	0.007
	Walking 1 h every day					−0.2085	0.071	−0.35–−0.07	0.003
	AIC	5219.73				4941.48			

**Table 3:** Results of the Tobit regression analyses with individual 5-year cumulative outpatient and medication expenditure as the dependent variable sorted into medication status groups.

Dependent Variable	Independent Variables	Taking Medication				Not Taking Medication			
		B	SE	95%CI	*p*-Values	B	SE	95%CI	*p*-Values
Cumulative medical expenditure	Intercept	8.8792	0.5495	7.80–9.96	0.000	7.3699	0.5202	6.83–8.40	0.0000
	Age	0.0389	0.0089	0.02–0.06	0.000	0.0538	0.0088	0.04–0.07	0.000
	Sex	−0.0283	0.0957	−0.22–0.16	0.767	−0.3597	0.1275	−0.60–−0.07	0.005
	Waist ratio	0.1301	0.0911	−0.05–0.31	0.153	0.0467	0.1292	−0.23–0.29	0.718
	Hypertension medication	−0.0031	0.0933	−0.19–0.18	0.973				
	Diabetes mellitus medication	0.4780	0.1244	0.23–0.72	0.000				
	Hyperlipidemia medication	0.1706	0.0907	−0.01–0.35	0.060				
	10 kg of weight gain since the age of 20	0.2232	0.0888	0.05–0.40	0.012	0.1836	0.1344	−0.07–0.05	0.172
	Walking 1 h every day	−0.1128	0.0854	−0.28–0.05	0.187	−0.3146	0.1131	−0.54–−0.10	0.005
	AIC	2254.55				2623.41			
